# Structural and physical determinants of the proboscis–sucking pump complex in the evolution of fluid-feeding insects

**DOI:** 10.1038/s41598-017-06391-w

**Published:** 2017-07-26

**Authors:** Konstantin G. Kornev, Arthur A. Salamatin, Peter H. Adler, Charles E. Beard

**Affiliations:** 1 Department of Materials Science and Engineering, Clemson University, Clemson, South Carolina 29634 USA; 2 Department of Aerohydromechanics, Kazan Federal University, Kazan, 420008 Russia; 3 Department of Plant and Environmental Sciences, Clemson University, Clemson, South Carolina 29634 USA

## Abstract

Fluid-feeding insects have evolved a unique strategy to distribute the labor between a liquid-acquisition device (proboscis) and a sucking pump. We theoretically examined physical constraints associated with coupling of the proboscis and sucking pump into a united functional organ. Classification of fluid feeders with respect to the mechanism of energy dissipation is given by using only two dimensionless parameters that depend on the length and diameter of the proboscis food canal, maximum expansion of the sucking pump chamber, and chamber size. Five species of Lepidoptera — White-headed prominent moth (*Symmerista albifrons*), White-dotted prominent moth (*Nadata gibosa*), Monarch butterfly (*Danaus plexippus*), Carolina sphinx moth (*Manduca sexta*), and Death’s head sphinx moth (*Acherontia atropos*) — were used to illustrate this classification. The results provide a rationale for categorizing fluid-feeding insects into two groups, depending on whether muscular energy is spent on moving fluid through the proboscis or through the pump. These findings are relevant to understanding energetic costs of evolutionary elaboration and reduction of the mouthparts and insect diversification through development of new habits by fluid-feeding insects in general and by Lepidoptera in particular.

## Introduction

Insects that feed on fluids have unique sucking mouthparts^1, 2^. Over the past 350 or so million years, fluid feeders have diversified to exploit different food sources including nectar, phloem, xylem, and cellular contents of plants, and carrion, dung, sweat, tears, urine, and blood of animals^3–5^. Ever since Darwin predicted that a sphinx moth with an extraordinarily long proboscis feeds from the equally long nectar spur of the orchid *Angraecum sesquipedale* ^6, 7^, feeding devices of insects have been a popular subject of evolutionary biology^3, 4, 8–11^. Evolution and diversification of insects and their feeding organs within the context of device morphology, properties, and functional performance are among the most attractive and demanding areas of study^12, 13^. Consideration of the organism as a hierarchical system with different levels of structure and activity requires identification of the mechanisms for division of labor and correlation between the structural units, and their adaptability to environmental changes^12–14^. This idea has been succesfully investigated for vertebrate feeding devices^15, 16^ but has not caught the attention of those investigating insect feeding devices.

Beginning with the seminal works of Bennet-Clark^17^, Tawfik^18^, and Kingsolver and Daniel^2, 19–21^, performance of insect fluid feeders has been evaluated on the basis of the proboscis^22, 23^. The sucking pump, which generates the suction pressure, was largely set aside in the structural hierarchy of insect feeding organs. Kingsolver and Daniel hypothesized that muscular energy of the insect is spent on combating viscous friction of fluid moving through the proboscis^20, 21^. This hypothesis allowed them to decouple the pump from the proboscis. However, physiological features of the pump cannot guarantee that viscous dissipation of moving fluid in the pump is always negligible; X-ray phase-contrast imaging experiments^24–26^ and neurophysiological analysis of the lepidopteran sucking pump^27^ revealed complex liquid flow through the pump, adding a degree of doubt to this assumption. Estimates of pressure generated by the sucking pump of Lepidoptera with long proboscises^28^ show that to defeat enormously high viscous dissipation during unidirectional flow of liquid through the food canal requires the insect to invoke other physiological and behavioral mechanisms.

Over evolutionary time, as insects came to inhabit nearly all terrestrial and freshwater habitats, fluid feeders adapted to feed from a variety of resources, probing into crevices, cavities, and pores to acquire liquids^29–31^. Insects can feed on thick, highly viscous liquids, such as honey, or on thin, almost inviscid mineral water^2, 22, 29–31^. Thus, in the evolutionary development of insect feeding organs, the variety of possible scenarios for structural-functional performance of the pump–proboscis pair cannot be ignored. Evolution of insects has involved both the increase and decrease of organ size^31–35^; accordingly, feeding devices of insects encompass a wide range of sizes, from those of extremely small insects such as aphids^36, 37^ to 20-centimeter long proboscises and powerful sucking pumps of some sphinx moths^7^. This large span of sizes is associated with different behavioral strategies and physical and materials organization of the feeding devices.

Flow in the proboscis and sucking pump during fluid uptake is interdependent. The geometry of sucking pumps of fluid-feeding insects is complex, many details are poorly understood, and quantitative morphological data are scarce^17, 27, 38–40^. To illustrate a range of sizes of the sucking pump, the proboscis, and their geometry, we selected four species, representing proboscises from 0.3 mm to about 70 mm long: White-headed prominent moth (*Symmerista albifrons*), White-dotted prominent moth (*Nadata gibosa*), Monarch butterfly (*Danaus plexippus*), and Carolina sphinx moth (*Manduca sexta*). We added a fifth representative from the literature^41^, the Death’s head sphinx moth (*Acherontia atropos*). These species demonstrate a general motif in the arrangement of feeding devices: although the pump is sizable relative to the head, proboscis length varies over a broad range of scales. Thus, the ratio of maximum size of the pump chamber to proboscis length changes from infinity to zero, suggesting that the mechanism of energy dissipation changes from one group of insects to another. We hypothesize that in insects with a small ratio of chamber size to proboscis length, energy dissipation should be associated with the viscous drag of liquid moving through the proboscis (“proboscis dissipation”), whereas in insects with a large ratio of chamber size to proboscis length, energy dissipation should come from the viscous drag of liquid on the moving pump plunger (“pump dissipation”).

Using a fluid-mechanics model of coupled laminar flow through the proboscis and pump, we examine this hypothesis. This model provides a relationship between displacement of a movable plunger and average pressure, as well as the force required to expand the pump chamber. The results allow quantitative classification of the role of different feeding parts, emphasizing division of labor between them, as well as their integration.

## Materials and Methods

The two species of prominents were collected at an ultraviolet light in Clemson, SC, on 26 August 2016. Monarch butterflies and Carolina sphinx moths were laboratory-reared from pupae and larvae, respectively, obtained as stock from Shady Oak Butterfly Farm (Brooker, FL) and Carolina Biological Supply Co. (Burlington, NC), respectively.

Specimens were prepared for scanning electron microscopy by fixing them in 80% ethanol. The head of each specimen was removed and obscuring brushes of scales were excised with microscissors. Each head was dehydrated through an ethanol series to 100%, chemically dried with hexamethyldisilazane, mounted on a conductive stub with double-sided adhesive conductive tape, sputter coated with platinum for 3–4 min, and imaged with a Hitachi TM-3000 Scanning Electron Microscope (composite mode, 15 kV, and full vacuum). Proboscis measurements were made from scanning electron micrographs.

For micro-computed tomography (micro-CT) imaging, *M*. *sexta* was dispatched by freezing at −76 °C, whereas, *D*. *plexippus*, *N*. *gibosa*, and *S*. *albifrons* were killed at −80 °C and imaged within 1 hour. Scans were made at North Dakota State University Electron Microscopy Core Lab in Fargo, North Dakota. Each sample was placed at the top of a Kapton 40 tube with the head protruding. CT scans were collected using a GE Phoenix v|tome| x s X-ray computed tomography system (micro-CT) equipped with a 180-kV high power nanofocus X-ray tube xs|180nf and a high-contrast GE DXR250RT flat panel detector. One thousand projections of the sample were acquired at a voltage of 60 kV and a current of 240 μA, using a molybdenum target. Detector timing was 1500 ms and total acquisition time was 1 hour and 40 minutes. Sample magnification was 55.22x with a voxel size of 3.62 μm. The acquired images were reconstructed into a volume data set, using GE datos|x 3D-computer tomography software version 2.2. The reconstructed volume was viewed and manipulated using VGStudio Max MyVGL viewer version 3.0 by Volume Graphics. Images were saved at 3000 DPI, and measurements of the pump were taken using Fiji ImageJ (http://imagej.net/Citing).

## Results

### Anatomical features of the sucking pump and proboscis

**Figure 1:**
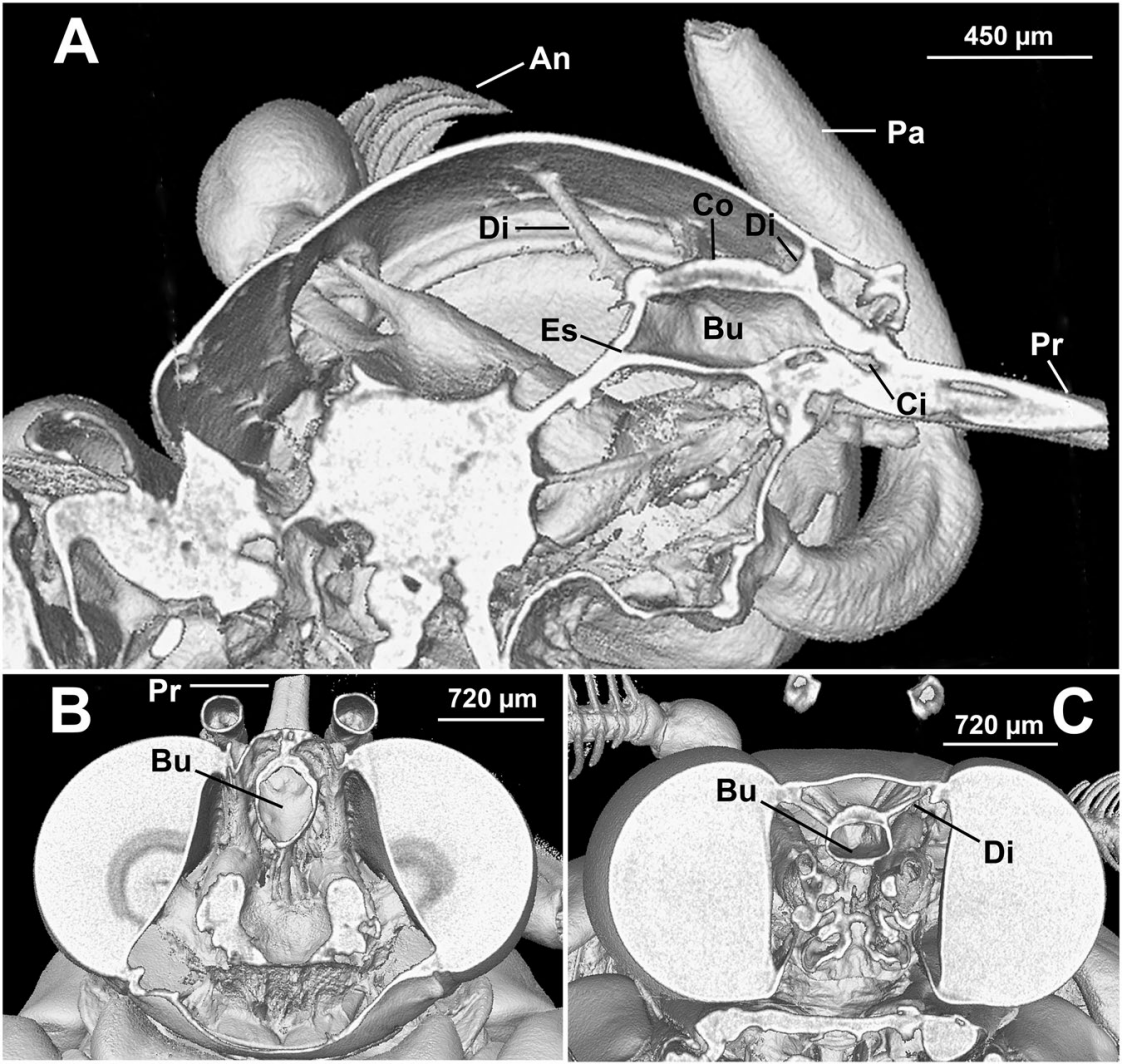
Micro-CT images of *Nadata gibosa*, showing relative position of the sucking pump in the head. (**A**) Lateral view. (**B**) Dorsal view. (**C**) Anterior view. An, antenna; Bu, buccal chamber; Ci, cibarium; Co, compressor; Di, dilator muscles; Es, esophagus; Pa, labial palp; Pr, proboscis.

We adopt the terminology used by Davis & Hildebrand^27^ to describe the two-part sucking pump of adult Lepidoptera (Fig. 1), which is composed of an anterior cibarium that leads into a larger, posterior buccal chamber. Paired sets of dilator muscles lead from the head capsule to the flexible roof (plunger) of the sucking pump, passing through buccal compressor muscles that envelop the entire dorsum and anchor at the dorsoventral junction of the well-sclerotized pump floor; closure of a posterior valve allows negative pressure to form in the pump as the dilators contract, thereby raising the plunger and bringing liquid from the food canal of the proboscis into the pump, whereas closure of an anterior (oral) valve allows swallowing when the dilators are relaxed and the compressors contract^42^.

**Figure 2:**
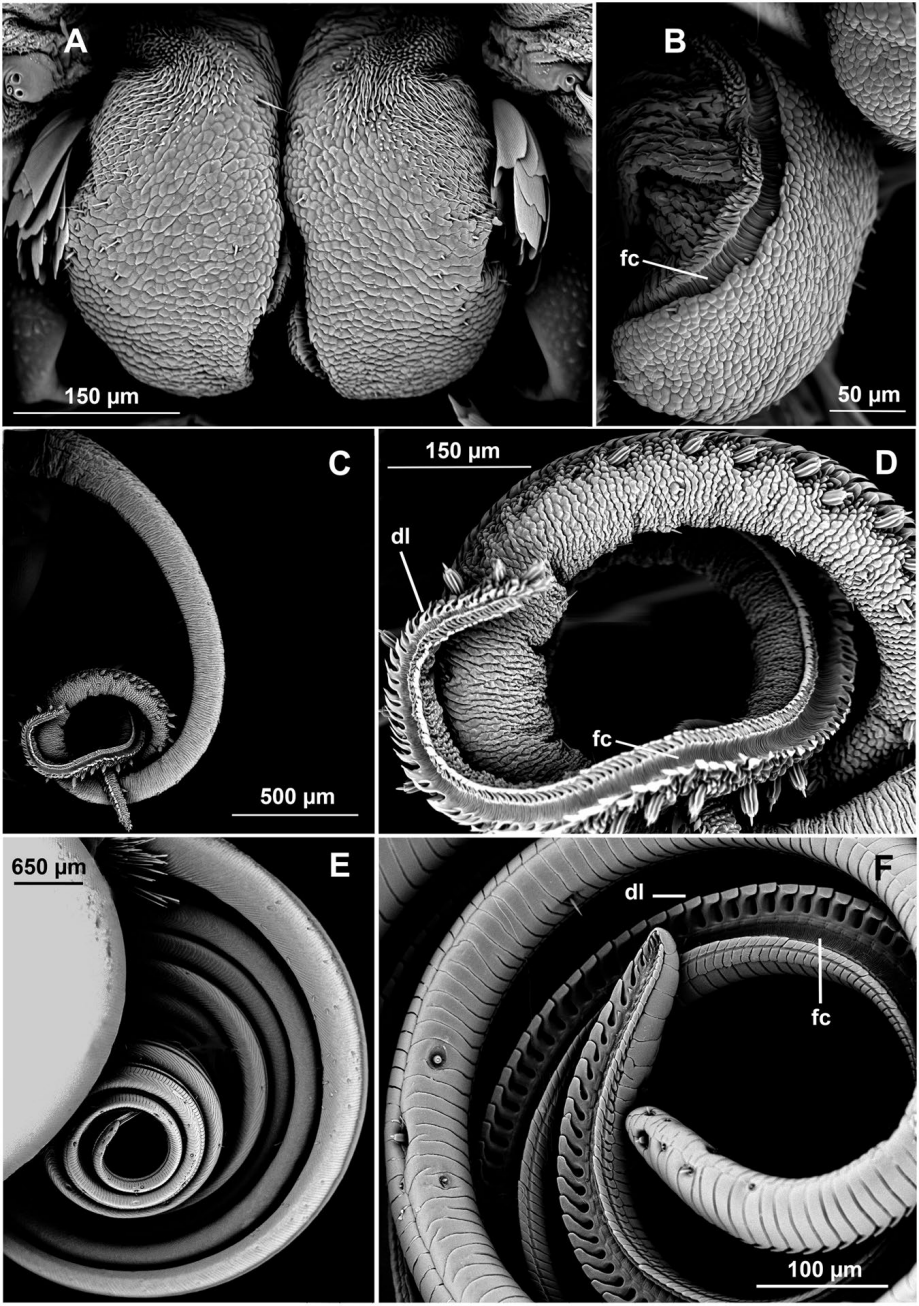
Scanning electron micrographs of moth proboscises representing various lengths: short, intermediate, and long. (**A**,**B**) *Symmerista albifrons*, anterior and medial views, respectively. (**C**,**D**) *Nadata gibosa*, lateral and apicolateral views, respectively, (**E**,**F**) *Manduca sexta*, lateral and apicolateral views, respectively. In (**C**–**F**), the galeae have been separated apically to show the food canal (fc) and dorsal legulae (dl).

Variation among species was apparent, most conspicuously in proboscis length and configuration (Fig. 2). *Manduca sexta* had a proboscis up to 200 times longer than that of *S*. *albifrons*. The proboscises of *D*. *plexippus*, *A*. *atropos*, and *N*. *gibosa* were intermediate in length. The galeae of all species except *S*. *albifrons* were semicylindrical and linked via legulae, forming a closed food canal. The galeae of *S*. *albifrons* were dorsoventrally compressed, presenting a more flattened proboscis without legulae; although the galeae were not linked, a food canal with sensilla was present along the medial margin of each galea.

Although the general structure of the pump (Fig. 1), including presence of three sets of dilators, was similar among species, dimensions of the pump varied among species (Table 1). The pump was wider than long in *M*. *sexta* and *S*. *albifrons*, but longer than wide in the other species. The greatest dimensions were in *A*. *atropos* and smallest in *S*. *albifrons*.

**Table 1:** Characteristics of the proboscis and sucking pump of selected Lepidoptera. *f* is a dimensionless factor that specifies the mechanism of energy dissipation.

Species	Proboscis length, ${l}_{p}$ (mm)	Food canal diameter, *d*_*p*_ (µm)	Pump length^b^, L (µm)	Pump width^c^, W(µm)	Pump height^d^, *h* (µm)	${\boldsymbol{f}}=\frac{{{\boldsymbol{l}}}_{{\boldsymbol{p}}}{{\boldsymbol{h}}}^{3}}{{{\boldsymbol{d}}}_{{\boldsymbol{p}}}^{4}}$	*g* = *d*_*p*_/*L*
*Acherontia atropos* ^a^	10.1	494.6	2417.6	2030.3	765.9	75.8	0.2
*Danaus plexippus* ^e^	14.4	35.0	886.2	606.3	145.6	29519	0.03
*Manduca sexta* ^e^	50–70	82.5	1834.5	2272.2	1227.4	1995780	0.04
*Nadata gibosa*	3.58	25.4	598.3	362.8	240.4	119495	0.04
*Symmerista albifrons* ^f^	0.35	17.3	298.8	358.8	91.0	NA	0.12

^a^Data from Fig. 2D,E in ref. 41 were analyzed in Image J.

^b^Longest distance within the chamber, parallel to the floor of the pump.

^c^Greatest width within the chamber, perpendicular to the length of the pump chamber.

^d^Greatest height, dorsal to ventral, along the mid-sagittal plane and perpendicular to the length of the pump.

^e^Proboscis lengths for *D*. *plexippus* and *M*. *sexta* and food canal diameter for *D*. *plexippus* were taken from the literature^25, 66, 71^.

^f^Diameter of the food canal of a single galea, measured from dorsum to venter. The two galeae do not meet (Fig. 2A); therefore, a single food canal is not formed; the food canal was assumed to be circular.

### Simplified geometrical model of the pump

The sucking pump forms a functional complex with the proboscis. The pump brings liquid into the gut^43^, operating against forces imposed by a narrow fluid conduit, the food canal^25, 28^. Thus, to understand fluid uptake, the proboscis must be interpreted in the context of the sucking pump, although inferences can be made independently about the associated functionality of each. For instance, we suggest that a smaller pump (e.g., *S*. *albifrons*) indicates a shorter proboscis and less suction pressure, as the insect does not need to combat friction forces associated with fluid transport through a long proboscis. In contrast, we also suggest that greater proboscis volume (e.g., *M*. *sexta*) implies a stronger pump. To make a quantitative analysis of the efficiency of the pump–proboscis pair and elucidate the importance of their coupling, we need to simplify the geometry of the pump and food canal, while including the main features.

**Figure 3:**
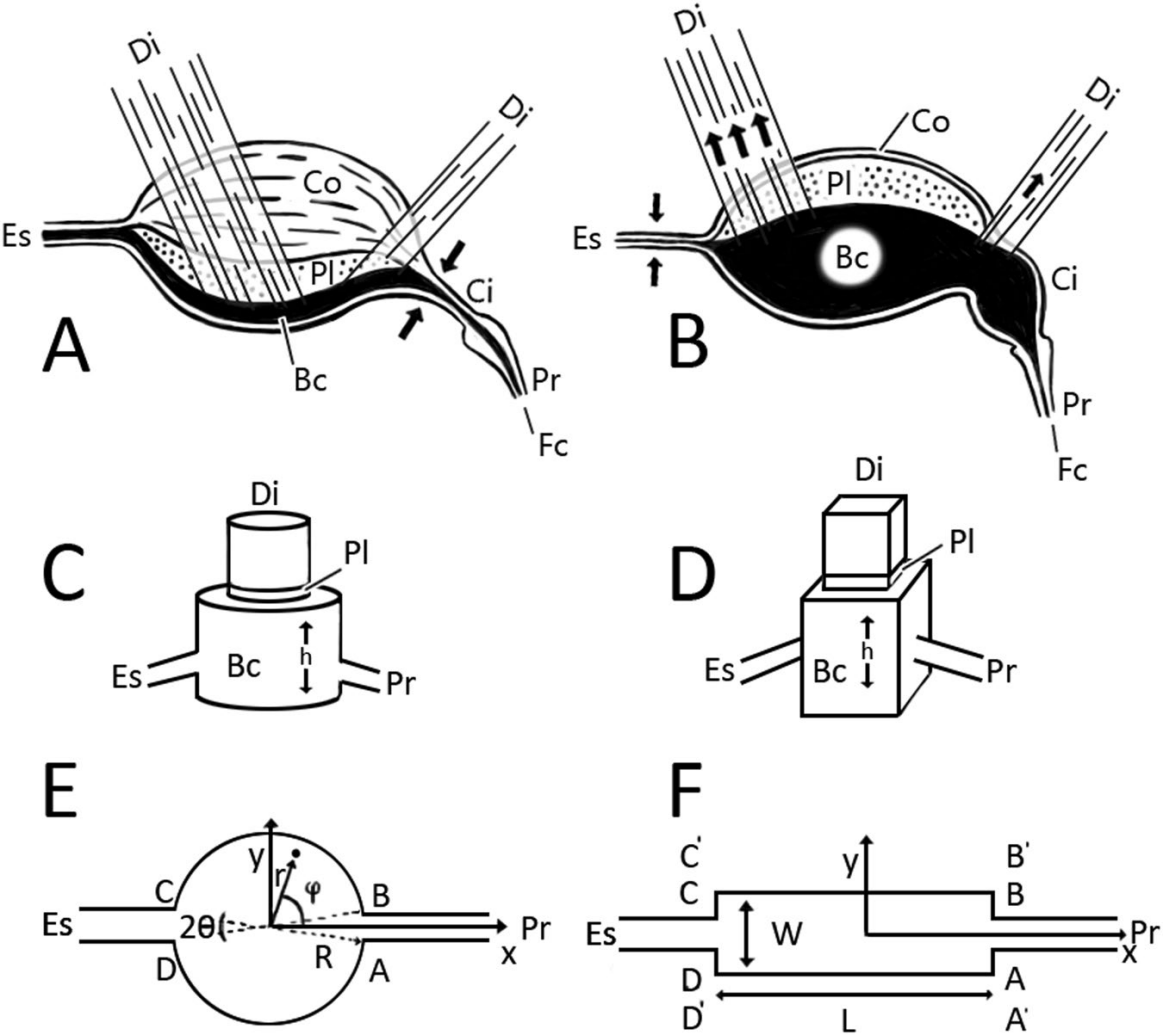
Schematic of lepidopteran sucking pump. (**A**,**B**) Lateral view of the sucking pump, consisting of the buccal chamber (Bc) and cibarium (Ci). Flow of liquid is from right to left, through the food canal (Fc) of the proboscis (Pr), into the sucking pump (Ci and Bc), and exiting via the esophagus (Es). Arrows indicate direction of muscle contraction. In (**A**), the dilator muscles (Di) have relaxed and the compressor muscle (Co) of the pump has contracted, forcing the plunger (Pl) toward the chamber floor. In (**B**), the compressor muscle of the pump has relaxed and the dilator muscles (Di) have contracted, drawing the plunger toward the dorsum of the buccal chamber. (**C**–**F**) Models of the sucking pump (not to scale). (**C**) The Daniel-Kingsolver model of a sucking pump. The buccal chamber is modeled as a cylindrical chamber and the circular plunger fits the chamber firmly. When the plunger moves in the vertical direction, it changes the expansion *h*. The proboscis and esophagus are attached to the buccal chamber. (**D**) The Bennet-Clark model of a sucking pump. The buccal chamber is modeled as a rectangular box and the plunger fits the box firmly. When the plunger moves in the vertical direction, it changes the expansion *h*. The proboscis and esophagus are attached to the buccal chamber. (**E**) Cirular (radius R) lengthwise cross-section of a model pump. Ratio AB/R is equal to 2*θ*. The model pump has opening AB connecting the chamber with the proboscis and opening CD connecting the chamber with the esophagus. (**F**) Rectangular lengthwise cross-section of width W and length L of a model pump. Any point on the chamber floor can be specified by either Cartesian coordinates (*x*,*y*) for rectangular U-chambers or by cylindrical coordinates (*r*,*φ*) for circular U-chambers.

Bennet-Clark was probably first to put forward a convenient geometrical model of the sucking pump^17^. He modeled the buccal chamber as a U-shaped dish covered by a piston (plunger) moving up and down through the central opening of the dish. In *Rhodnius prolixus* ^17^, the buccal chamber lengthwise is a long rectangular channel. The cibarium opens by a narrow aperture AB where it connects to the food canal of the proboscis. At the opposite side of this chamber is another aperture CD connecting the chamber with the esophagus (Fig. 3F). In the Bennet-Clark model^17^, the plunger is assumed to tightly fit the U-shaped floor. In the majority of cases, pump height in the *z*-direction perpendicular to the floor, *h*, is smaller than the other scales, L, W. For example, in *Rhodnius prolixus*, Bennet-Clark documented the following sizes: L ∼ 3–5 mm, W ∼ 0.28 mm, and maximum expansion of the buccal chamber *h* ∼ 0.16 mm.

For insects such as Lepidoptera and blood-sucking hemipterans, the U-shaped cross-section of the pump is the main geometrical motif^21, 44–46^. In our studied lepidopterans, we identified two characteristic shapes of the U-shaped dish-like buccal chambers: for one group of insects the lengthwise cross-section can be approximated by a rectangle^17^ and for another group of insects it can be approximated by a circle^21^. Mechanical action of the pump is captured quite well by the Bennet-Clark-Daniel-Kingsolver model (Fig. 3). This model has all the necessary functional mechanisms that are present in different insect species^21, 44–46^; yet, it is sufficiently simple and can be analyzed in detail to reveal the physical causes of liquid flow through the feeding devices of sucking insects. Hereafter, the chamber floor and plunger are assumed to be flat, so the chamber is a cylinder with rectangular or circular cross-section. The chamber roof and floor are assumed to be rigid.

### Flow model

When the plunger starts to move upward to expand the cavity or when it approaches the floor to close the chamber, the in-plane components of the velocity vector, *V*_*x*_ and *V*_*y*_, are much greater than the trans-plane velocity component *V*_*z*_. At the reported flow rate of *Q* ∼ 0.33 mm^3^/sec, longitudinal velocity in the pump of *Rhodnius prolixus* is approximately *V*_*x*_ ∼ *Q*/(*Wh*) ∼ 7 mm/sec^17^. Assuming that the blood is incompressible, like any liquid at the pressure of interest^47^, and using the mass balance, which shows that the amount of blood entering the chamber and moving in the longitudinal direction, *V*_*x*_ *Wh*, is approximately equal to the amount of liquid lifted up by the plunger in the *z*-direction, *V*_*z*_ *WL*, we confirm that in-plane velocity is much greater than trans-plane velocity, *V*_*x*_ ∼ (*L*/*h*)*V*_*z*_. The large parameter (*L*/*h*) makes the ratio *V*_*x*_/*V*_*z*_ about 30; for smaller openings this ratio is even greater. For Lepidoptera, the ratio *V*_*x*_/*V*_*z*_ is also large; at the point of maximum pump height it varies between 2 and 6 for the species in Table 1; for smaller expansions, this ratio is much greater^21, 28^. Thus, flow in the pump is almost two dimensional, with in-plane velocity dominating trans-plane flow, following plunger movement. Only one valve, either AB or CD, is opened during each cycle of liquid uptake (AB) or swallowing (CD) (Fig. 3E,F).

In fluid mechanics, a quasi-steady flow through narrow conduits, where in-plane velocity is much greater than trans-plane velocity, is called Hele-Shaw flow, named after its discoverer Henry Selby Hele-Shaw^48^. These flows are studied in relation to liquid lubrication of mechanical bearings, constituting lubrication theory^49^. In lubrication theory, the pressure through a conduit of thickness z is nearly independent of the z-coordinate as soon as the thickness of the liquid layer becomes much smaller than the longitudinal scale of flow. The in-plane flow is described by an average in-plane velocity ${\boldsymbol{V}}({V}_{x},{V}_{y})=(1/h){\int }_{0}^{h}{\boldsymbol{v}}(x,y,z)dz$, where the 2D local velocity ***v*** is a solution to the Stokes equation of fluid dynamics^49^. Arguments in favor of neglecting inertial terms in the full Navier-Stokes equations are as follows.

In physiological fluid mechanics, an important indicator of flow unsteadiness is the Womersley number^21, 44, 50, 51^, ${\rm{Wo}}=h\,\sqrt{2\pi f\rho /\eta }$, where *ρ* is the density of liquid, *η* is the dynamic viscosity of fluid, and *f* is the beating frequency of the pump. For small Womersley numbers, *Wo* < 1, unsteady effects can be neglected, and for large Womersley numbers, *Wo* > 1, these effects become important. Calculating the Womersley number for the pump of *Rhodnius prolixus*, for which Bennet-Clark reported *f* = 3 beats/sec^17^, and taking water as the reference for viscosity and density of blood, we see that *Wo* ∼ 3 · 10^3^ *h*, where *h* is measured in meters. Pump height cannot exceed *h*_*max*_ ∼ 0.16 · 10^−3^ m; hence, during the expansion–contraction cycle when pump height is much smaller than *h*_*max*_, the Womersley number is less than 1, implying that unsteady effects can be neglected for this insect. The same conclusion has been drawn for Lepidoptera^21^.

### Mathematical model

The Cartesian system of coordinates (*x*, *y*) is shown in Fig. 3F, with the center at the chamber floor.

The flow is described by velocity vector ***V***(*V*_*x*_, *V*_*y*_), which in lubrication theory is related to the pressure gradient as ref. 49

1 $$({V}_{x},{V}_{y})=(-\frac{{h}^{2}}{12}\frac{\partial {p}}{\partial {x}},-\frac{{h}^{2}}{12}\cdot \frac{\partial {p}}{\partial {y}}),$$

where *p*(*x*, *y*) is pressure at point (*x*, *y*) and *h* = *h*(*t*) is pump height at the given moment of time *t*. This vectorial equation emphasizes the strong quadratic dependence of velocity on pump height *h*: at the same pressure gradient, flow significantly slows down as gap thickness *h* decreases. For example, a threefold decrease of gap thickness results in nearly an order of magnitude decrease of velocity. Thus, as the plunger moves upward, expanding the pump chamber, velocity progressively increases and the chamber is filled faster. This effect has never been discussed in the context of feeding biomechanics and deserves special attention.

The local balance of mass for liquid filling the pump chamber is stated as

2 $$\frac{dh}{dt}+\frac{\partial (h{V}_{x})}{\partial {\rm{x}}}+\frac{\partial (h{V}_{y})}{\partial {\rm{y}}}=0.$$

Upon substitution of eq. (1), we obtain

3 $$\frac{12\eta }{{h}^{3}}\frac{dh}{dt}=\frac{{\partial }^{2}p}{\partial {x}^{2}}+\frac{{\partial }^{2}p}{\partial {y}^{2}},$$

In this equation, pump height *h*(*t*) depends only on time *t* and not on coordinates (*x*, *y*). Hence, eq. (3) is the Poisson equation with respect to the unknown pressure distribution *p*(*x*, *y*) and the left hand side is independent of (*x*, *y*).

Formulating the boundary conditions for eq. (3), it is sufficient to consider only the uptake stroke when the plunger moves upward and hence *dh*/*dt* > 0. Fluid injection into the esophagus follows a similar flow scheme, with the sign change, *dh*/*dt* < 0. We will comment on flow differences when necessary.

The first set of boundary conditions for eq. (3) states that the sides ADCB are impermeable to liquid, ***V*** · ***n*** = 0, where ***n*** is the outward unit normal vector. Expressing velocity components (*V*_*x*_, *V*_*y*_) through the pressure gradient, eq. (1), we have

4 $${\boldsymbol{\nabla }}p\cdot {\boldsymbol{n}}=0\,\quad {\rm{at}}\,{\rm{ADCB}}.$$

The second set of boundary conditions connects flow in the proboscis with flow in the pump chamber. Considering the food canal in the proboscis as a cylindrical tube and assuming the Womersley number is small, *Wo* < 1, we can model flow in the food canal as Hagen-Poiseuille flow^2, 17–21, 28^ for which pressure through the cross-section of the food canal does not change. Thus, pressure at entrance AB must be equal to pressure in the food canal of the proboscis,

5 $$p={{\rm{P}}}_{p}(t)\,{\rm{at}}\,AB.$$

If the plunger position as a function of time *h*(*t*) is known and the pressure at the cibarial entrance, P_*p*_(*t*) is known, then model (2)–(5) completely describes flow in the pump chamber. In the next section, we show how these two functions *h*(*t*) and P_*p*_(*t*) are related.

### Coupling flow in the proboscis with change in pump volume

The sucking pump of insects in the majority of cases works as a displacement pump and, hence, depends on expansion of the chamber indissolubly connected to the elastic recoil of supporting muscles^44, 45^. Consider a stroke associated with filling the sucking pump with liquid. During this stroke, esophagus entrance CD remains closed (Fig. 3E,F). Integrating eq. (2) over the area of the pump chamber and applying the Gauss theorem, we have

6 $$\frac{dh}{dt}{\rm{A}}+{\iint }^{}\nabla \cdot (h{\boldsymbol{V}})dA=\frac{dh}{dt}{\rm{A}}+{\oint }^{}h{\boldsymbol{V}}\cdot {\boldsymbol{n}}dS=\frac{dh}{dt}{\rm{A}}-\frac{{h}^{3}}{12\eta }\overline{(\nabla {\rm{p}}\cdot {\boldsymbol{n}})}=0,$$

where

$$\frac{{h}^{3}}{12\eta }\overline{(\nabla {\rm{p}}\cdot {\boldsymbol{n}})}=-\frac{{h}^{3}}{12\eta }{\int }^{}\nabla {\rm{p}}\cdot {\boldsymbol{n}}dS={h}{\int }^{}({\bf{V}}\cdot {\boldsymbol{n}})dS=Q.$$

where $A=LW\,or$ $A=\pi {R}^{2}$ is the cross-sectional area of the chamber, and the integral average ${\int }^{}({\bf{V}}\cdot {\boldsymbol{n}})dS$ is taken over patch *AB*, ***n*** is the outward unit normal vector, dS is the surface element, and *Q* is the total discharge per unit time through the food canal. Finally, we have

7 $$Q=-A\frac{dh}{dt}.$$

This equation expresses the conservation of liquid volume, stating that the amount of liquid taken by the pump chamber (term on the right) is exactly equal to the amount of liquid passed through the proboscis (term on the left). This equation, therefore, mathematically defines the functional mechanism underlying the work of the displacement pump.

We model the food canal as a cylindrical tube of diameter *d*_*p*_ and length *l*_*p*_. Because the Womersley number is small, we can safely apply the Hagen-Poiseuille equation to relate the *total discharge* through these tubes with pressure P_*p*_(*t*) at AB. Pressure will be measured with respect to atmospheric pressure so that P_*p*_(*t*) = 0 implies that the pressure is atmospheric. We, therefore, have the following linear relationship between *Q* and P_*p*_(*t*):

8 $$Q=\,\frac{\pi {d}_{i}^{4}}{128\eta }\frac{{{\rm{P}}}_{p}(t)}{{l}_{p}},$$

Replacing the left side of Eq. (7) with Eq. (8), one deduces

9 $${{\rm{P}}}_{p}(t)=-A\frac{128\eta {l}_{p}}{\pi {d}_{p}^{4}}\frac{dh}{dt}.$$

Thus, pressure generated at opening AB is directly proportional to the rate of volume expansion of the pump chamber. At the same rate of expansion (positive *dh*/*dt*), the larger the chamber area *A*, the greater the suction pressure (negative P_*p*_). This equation closes the mathematical formulation of the problem for flow in the sucking pump and sets an important scale for pressure at the chamber entrance.

### Rate of pump expansion sets the scale for pressure distribution

The linearity of Eq. (3) suggests that pressure *p* and velocity of plunger movement *dh*/*dt* are linearly connected. Introducing dimensionless coordinates, taking the side of the rectangular pump chamber, *l*_*r*_ = *L*, or the radius of a circular chamber, *l*_*c*_ = *R* as a “yardstick”, (*x*/*L*, *y*/*L*) → (*X*,*Y*) we see that the pressure scales as $p\propto (12\eta {l}_{i}^{2}/{h}^{3})(dh/dt)$, i = r, c; hence, we can define the pressure as

10 $$p=(\frac{12\eta {l}_{i}^{2}}{{h}^{3}})(\frac{dh}{dt})\,P(X{,}Y){,}\,i={\rm{r}}{,}{\rm{c}}{,}$$

where *P*(*X*, *Y*) is the dimensionless pressure and subscript “r” stands for a rectangular and “c” for a circular pump chamber, i.e. *l*_*r*_ = *L* and *l*_*c*_ = *R*. For the dimensionless pressure *P*(*X*, *Y*), eq. (3) is rewritten as

11 $$\frac{{\partial }^{2}P}{\partial {X}^{2}}+\frac{{\partial }^{2}P}{\partial {Y}^{2}}=1{,}$$

and the boundary conditions (4) and (5) take on the form

12 $${\boldsymbol{\nabla }}P\cdot {\boldsymbol{n}}=0\,{\rm{at}}\,ADCB$$

13 $$P={{\rm{K}}}_{i},\,\,{\rm{at}}\,AB,$$

where

14 $${{\rm{K}}}_{i}=-\frac{32{h}^{3}{l}_{p}}{3{l}_{i}^{2}\pi {d}_{p}^{4}}{A}_{i}{,}\quad i={\rm{r}}{,}{\rm{c}}.$$

These scaling arguments allow us to deduce the functional dependence of pressure generated by the pump on physiological parameters of the insect,

15 $$p=\,(12\eta {l}_{i}^{2}/{h}^{3})(dh/dt)\,P(X{,}Y{,}{{\rm{K}}}_{i}{,}\frac{|AB|}{{l}_{i}}),\quad i={\rm{r}}{,}{\rm{c}}.$$

For a rectangular pump chamber the dimensionless pressure *P* also depends on the aspect ratio L/W. In dependence (15), the dimensionless pressure *P* is a solution to the boundary-value problem (11)–(14). This solution does not depend on rate of expansion of the pump chamber, but depends only on height at time, *h*(*t*), through parameter K_*i*_. Flow generated by this pressure *P* has many common features with Hele-Shaw flow in thin films^48, 49^. This connection has important physiological consequences discussed below. The definition of (15) can be detailed further as shown below.

## Flow analysis

### Analogy with Hele-Shaw flow

Hele-Shaw experimented with a flow cell made of two large parallel plates with a certain gap between them; liquid was forced to move through the gap parallel to the plates by applying a pressure differential between two openings^48, 49^. In the case of a sucking-pump chamber, liquid is moved by the plunger, and pressure at the chamber openings is built on its own. Nevertheless, the effect of the moving plunger can be modeled in a Hele-Shaw cell by adjusting the applied pressure distribution as follows. The dimensionless pressure *P*(*X*, *Y*) can be represented as a superposition of two pressure distributions: *P* = ${P}_{{\rm{squeeze}}}$ + ${\mathop{P}\limits^{ \sim }}_{i}$. *i* = *r*, *c*. The first is given as ${P}_{{\rm{squeeze}}}=\frac{1}{2}{(x/L)}^{2}-\frac{1}{8}+{K}_{r}$ for a rectangular chamber and ${P}_{{\rm{squeeze}}}=\frac{1}{4}{(r/R)}^{2}-\frac{1}{4}+{K}_{c}$ for a circular chamber. These pressures ${P}_{{\rm{squeeze}}}$ satisfy Eq. (11) and boundary condition (13) but boundary condition (12) is not satisfied. Looking at the definition of dimensional pressure (10), one infers that pressure $p=(12\eta {l}^{2}/{h}^{3})(\frac{dh}{dt}){P}_{{\rm{squeeze}}}\,\,$ is caused by volumetric squeezing of liquid from the chamber. Thus, the dimensionless pressure ${P}_{{\rm{squeeze}}}$ does not describe real flow in the sucking pump since it allows liquid to penetrate the pump walls. To correct it, one has to introduce a new unknown pressure that satisfies eq. (11) with the zero right-hand side. The boundary conditions for this new pressure have to be corrected. Thus, representing flow as a superposition of these two flows, *P* = ${P}_{{\rm{squeeze}}}$ + ${\mathop{P}\limits^{ \sim }}_{i}$. *i* = *r*, *c*, the total dimensionless pressure distribution in the chambers is written as

16 $$\begin{array}{c}{Rectangular}\,{chamber}\\ P{=}\,[\frac{{1}}{{2}}{(\frac{{x}}{{L}})}^{{2}}{-}\frac{{1}}{{8}}+{\tilde{{P}}}_{{r}}(X{,}Y{,}\frac{{AB}}{{L}}{,}\frac{{W}}{{L}})]{+}{{K}}_{{r}}{,}\end{array}$$

17 $$\begin{array}{c}Circular\,chamber\\ P=[\frac{1}{4}{(\frac{r}{R})}^{2}-\frac{1}{4}+{\tilde{P}}_{c}(X{,}Y{,}\theta )]+{K}_{c},\end{array}$$

where the functions ${\tilde{P}}_{i}(X{,}Y)$ are the solutions for the following boundary-value problems

18 $$\begin{array}{c}{Rectangular}\,{chamber}\\ \frac{{\partial }^{2}{\tilde{P}}_{r}}{\partial {X}^{2}}+\frac{{\partial }^{2}{\tilde{P}}_{r}}{\partial {Y}^{2}}=0.\end{array}$$

19 $$\frac{\partial {\tilde{P}}_{r}}{\partial {\rm{Y}}}=0,{\rm{at}}\,C^{\prime} B^{\prime} \,{\rm{and}}\,A^{\prime} D^{\prime} ,{\frac{\partial {\tilde{P}}_{r}}{\partial {\rm{X}}}|}_{X=\pm \frac{1}{2}}=-\frac{1}{2}\,{\rm{at}}\,C^{\prime} D^{\prime} \,{\rm{and}}\,AA^{\prime} \,{\rm{and}}\,BB^{\prime} ,$$

20 $${\tilde{P}}_{r}=0{,}\,{\rm{at}}\,AB.$$

21 $$\begin{array}{c}Circular\,chamber\\ (\frac{1}{\tilde{r}}\,\frac{\partial }{\partial \tilde{r}}(\tilde{r}\frac{\partial {\tilde{P}}_{c}}{\partial \tilde{r}})+\frac{1}{{\tilde{r}}^{2}}\frac{{\partial }^{2}{\tilde{P}}_{c}}{\partial {\phi }^{2}})=0\end{array}$$

22 $$\frac{\partial {\tilde{P}}_{c}}{\partial \tilde{r}}=-\frac{1}{2}\,{at}\,BCDA{,}\tilde{r}=1{,}\quad \theta  < \phi  < 2\pi -\theta ,$$

23 $${\tilde{P}}_{c}(1{,}\phi )=0,\,{at}\,AB,\,\tilde{r}=1{,}\quad -\theta  < \phi  < \theta .$$

The boundary-value problems (18)–(23) are interpreted as some Hele-Shaw flow between fixed plates caused by some distributed injection/suction means to allow liquid flow through the cell boundaries when another part of the cell boundary is kept at zero pressure. From a set of physiological parameters, only the ratios AB/L = *g* and W/L, and the angle *θ*, enter the boundary-value problems (18)–(23) for various geometries. Therefore, the Hele-Shaw analogy allows one to significantly simplify parametric analysis of pressure distribution in the chambers.

Going back to the dimensional variables, one writes

24 $$\begin{array}{c}{Rectangular}\,{chamber}\\ p=\eta (dh/dt)\{(12/{h}^{3}){L}^{2}[\frac{1}{2}{(\frac{x}{L})}^{2}-\frac{1}{8}+{\tilde{P}}_{r}(X{,}Y{,}\frac{AB}{L}{,}\frac{W}{L})]-\frac{128WL{l}_{p}}{\pi {d}_{p}^{4}}\,\}\end{array}$$

25 $$\begin{array}{c}Circular\,chamber\\ p=\eta (dh/dt)\{(12/{h}^{3}){R}^{2}[{(r/R)}^{2}/4-1/4+{\tilde{P}}_{c}(X{,}Y{,}\theta )]-\frac{128{R}^{2}{l}_{i}}{{d}_{i}^{4}}\}\end{array}$$

The derived form of pressure distribution in the pump chamber (24) and (25) allows one to conclude that pressure in the chamber is *directly proportional to the rate of chamber expansion*/*contraction*; however, its dependence on gap thickness (*h*) is nonlinear. Pressure in the chamber is highly inhomogeneous and it is, therefore, instructive to evaluate the non-uniform part of the pressure distribution inside the chamber, which is described by the bracketed terms of eqs (24) and (25). These terms do not depend on time or proboscis length; hence, they can be evaluated once and used for further analysis of forces developed by the sucking pump.

### Pressure distributions in rectangular and circular chambers

**Figure 4:**
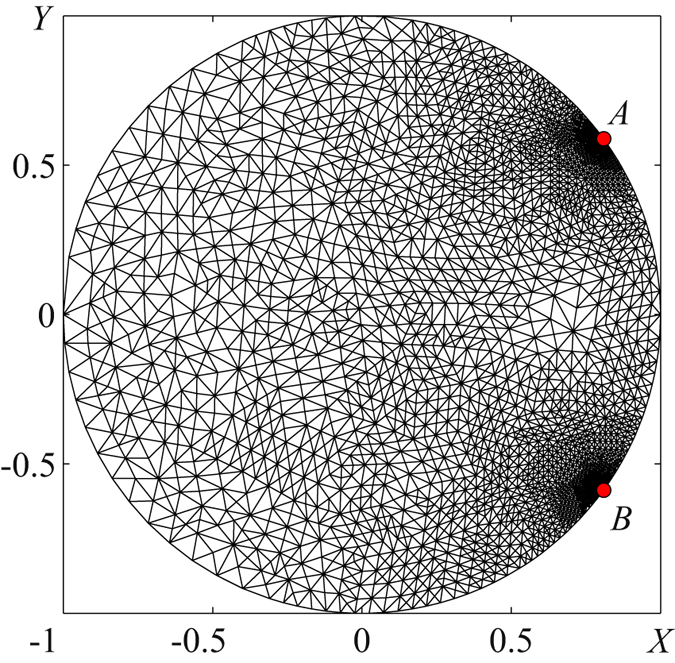
Example of FEM mesh used for pressure calculations. Each triangular finite element provides three pressure values associated with three nodes. The nodes are concentrated in the vicinity of points A and B where boundary conditions change. In this example, opening AB was 2*θ* = 2*π*/5, and 25 refinements were used. Dense nodes indicate high pressure gradients, whereas sparse nodes indicate lower gradients.

The boundary-value problems (18)–(23) were solved numerically by the Finite Element Method (FEM) implemented in Matlab R2010b PDE Toolbox. Pressure $\tilde{P}$ was determined at the triangle apexes distributed over the pump chamber. Figure 4 illustrates the mesh for a circular configuration. In the vicinity of points A and B where the pressure gradients are large, the number of nodes was purposely increased. Adaptive mesh refinement algorithms, implemented in MatLab, allow automatic mesh refinement so that for each simulation condition, mesh points are concentrated in areas of high-pressure gradients. Normally, 25 refinements provide appropriate accuracy of calculations.

**Figure 5:**
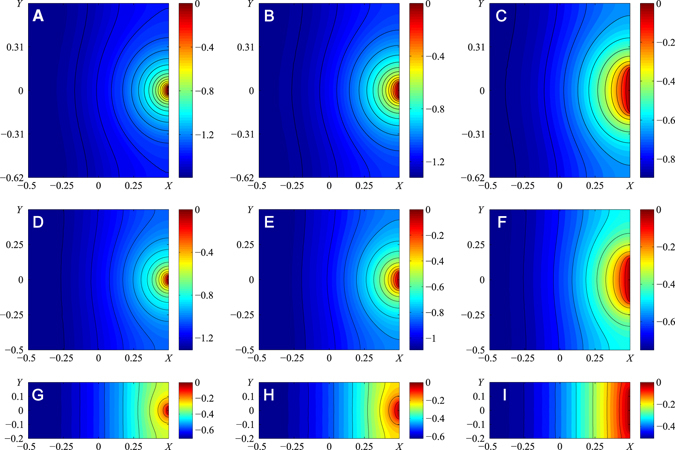
(**A**–**I**) Dimensionless pressure distribution *P* – *K*_*r*_ for three different sizes of food canal diameter to chamber length ratios |*AB*|/L and three different sizes of chamber elongations W/L. Parameter W/L is equal to (1.24, 1, 0.4) for the three rows from top to bottom, and parameter |*AB*|/L is equal to (0.05, 0.1, 0.3) for the three columns from left to right. The color bar sets the pressure level. Black lines indicate constant pressure; the two nearest lines have a dimensionless pressure difference of 0.08.

Three representative examples of dimensionless pressure distributions expressed by the bracketed terms in eqs (16) and (17) are shown in Fig. 5. To satisfy eqs (20) and (23), the pressure at opening AB where the food canal merges with the cibarial chamber is set at zero. The contour lines are solid in the figures and represent isobars. As expected, a significant change of pressure is found in the vicinity of entrance AB where the isobars are significantly bent to reflect the need for fluid velocity to abruptly change its direction: the velocity vector at opening AB has to be perpendicular to line AB, while the velocity vector just outside opening AB has to be parallel to chamber wall ADCB. Thus, opening AB causes pressure non-uniformity.

**Figure 6:**
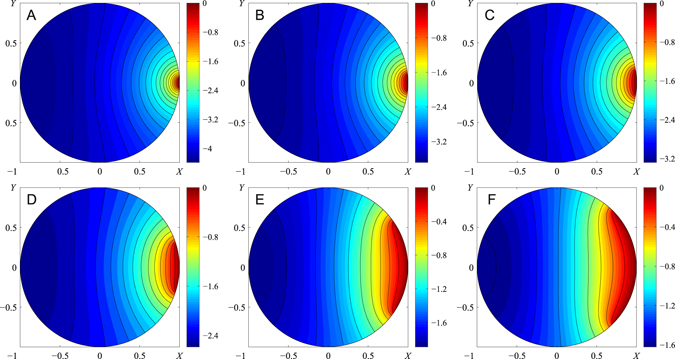
Dimensionless pressure distribution *P* − *K*_*c*_ for different ratios of the food canal diameter |AB| to chamber diameter 2 R. (**A**) |*AB*|/(2*R*) = 0.05, (**B**) |AB|/(2R) = 0.01, (**C**) |*AB*|/(2*R*) = *θ* = 0.15, (**D**) |AB|/(2 R) = 0.3, (**E**) |*AB*|/(2*R*) = *θ* = 0.6, (**F**) |*AB*|/(2*R*) = *θ* = 0.78. The color bar sets the pressure level. Black lines indicate constant pressure; the two nearest lines have a dimensionless pressure difference of 0.2.

As one moves along the X-axis (Fig. 5), pressure changes gradually from zero at entrance AB to maximum suction pressure at the outmost distal region, without any abrupt changes in pressure. The effect of chamber width to length ratio is shown in Fig. 5. Keeping the same ratio of diameter of the food canal, |AB|, to maximum length of the pump chamber, L, one observes that an insect with a square chamber develops stronger suction pressure at the distal side near the closed esophagus (Fig. 5). A circular chamber provides even stronger suction pressure at the side of the chamber opposite opening AB (Fig. 6). In this respect, insects with a circular chamber floor perform better with respect to building up stronger suction pressure at the same reasonable range of food-canal openings.

Comparison of Figs 5 and 6 also suggests that non-uniformity of pressure distribution is more pronounced in circular chambers: the isobars are curved significantly. Only about 70% of the rectangular and square areas lengthwise are engaged in setting up the pressure gradient: the isobars are curved in these regions. In the remaining 30% of the distal region lengthwise, the isobars are almost straight, implying that the chamber floor and movable plunger are subject to almost constant negative pressure. Moreover, as proboscis diameter |AB| approaches chamber width W, flow non-uniformity diminishes: the isobars straighten out, implying a significant reduction of the Y-component of the velocity vector. Thus, flow approaches unidirectional movement when fluid entering the chamber moves to the opposite side toward the esophagus parallel to the impermeable walls shown as A’D’ and B’C’ in Fig. 3F.

In contrast, circular chambers are more involved in pressure distribution over the whole chamber floor, even in chambers with the same |AB|/L = |AB|/(2 R) ratios, the isobars in a circular chamber are more bent, and the lengthwise span of the zone where the isobars are curved is always greater in a circular chamber. There is no universal tendency in identification of the zone of almost constant negative pressure; this zone significantly depends on the |AB|/R ratio and is shaped as a semi-circular segment in Fig. 6.

For both chamber geometries, the plunger movement leading to chamber expansion results in generation of negative pressure at the chamber periphery, closer to the esophagus. In the model, the cross-section of the pump opening correlates with plunger deformation. However, in insects, different sets of muscles power the food-canal opening and plunger movement^27, 41, 43^. With the calculated pressure distribution in Figs 5 and 6, one observes that insects are able to significantly change suction pressure by independently changing the size of the food canal opening at the cibarial entrance. In square and circular chambers, pressure changes almost twice with the ten-fold change of opening |AB|. This “frozen” picture of pressure distribution does not consider the change of the pressure scaling factor in eqs (24) and (25). This scaling factor changes when the plunger is pulled by dilator muscles. Thus, the general picture of pressure generation might be more complex, as the insect has more degrees of freedom to control pressure during the liquid uptake stroke. Nonetheless, the calculations reveal important material components influencing pump architecture in evolutionary developments of insects.

## Force acting on pump plunger and classification of energy (proboscis vs pump) dissipation mechanisms

### The forces

To evaluate performance of the dilator muscles that move the pump plunger to expand and contract the chamber, one requires estimates of resistive forces exerted on the plunger by the moving fluid. Total force *F*(*t*) acting on the pump plunger is calculated using eqs (24) and (25), and integrating pressure over the surface area of the plunger. The resulting equations have the same structure for both geometries:

26 $$\begin{array}{c}{Rectangular}\,{chamber}\\ {F}_{r}=(12\eta /{h}^{3})(dh/dt){L}^{4}[{C}_{r}-\frac{1}{12}]\frac{W}{L}-(dh/dt)\frac{128\eta {l}_{p}}{\pi {d}_{p}^{4}}{(LW)}^{2},\end{array}$$

where constant *C*_*r*_ depends only on the ratios W/L, and |AB|/L and is defined as

27 $${C}_{r}={\int }_{-W/2L}^{W/2L}{\int }_{-1/2}^{1/2}{\tilde{P}}_{r}dXdY,$$

28 $$\begin{array}{c}Circular\,chamber\\ {F}_{c}(t)=\,(12\eta /{h}^{3})(dh/dt){R}^{4}[{C}_{c}-\frac{\pi }{8}]-(dh/dt)\frac{128\eta \pi {l}_{p}}{{d}_{p}^{4}}{R}^{4},\end{array}$$

where the constant *C*_*c*_ depends only on |AB|/R and is defined as

29 $${C}_{c}={\int }_{-\pi }^{\pi }{\int }_{0}^{1}{\tilde{P}}_{c}rdrd\phi ,$$

In equations (26) and (28), functional dependence of force on velocity of pump expansion, *dh*/*dt* and position of the pump roof, *h*(*t*), is the same; only the constants for rectangular and circular chambers differ from one another. These equations support the assumption that the functional form of force dependence on materials parameters of the organism should not depend significantly on the choice of geometrical model for the chamber.

The first term in eqs (26) and (28) resembles the Stefan law of adhesion^52, 53^. The Stefan law states that detachment/attachment of closely positioned objects separated by a layer of a viscous fluid requires a force that is directly proportional to the velocity of the detaching/attaching article and inversely proportional to layer thickness cubed. The same law explains, for example, the ability of marine gastropods to attach themselves to a variety of substrates^54^. This term also emphasizes the importance of fluid viscosity; for example, *Acherontia atropos* feeding on honey in bee colonies should be able to generate a force about 10–100 times greater than that of *Manduca sexta* feeding on nectar, which is 10–100 times less viscous than honey^55, 56^. Paradoxically, as recently described^41^, the head anatomy including musculature and pump design largely follows that of Lepidoptera in general^1^ without any additional features to combat these viscous forces.

This paradox brings attention to the second term in eqs (26) and (28). This term shows that the liquid column attached to the liquid layer in the pump always opposes the muscular force acting on the plunger. For example, in the suction stroke, when the chamber expands, *dh*/*dt* > 0, one has to apply a force greater than the Stefan force to suck up the liquid from the proboscis. In the injection stroke, when the plunger squeezes liquid to the esophagus, *dh*/*dt* < 0, the liquid column in the esophagus resists movement of the pump roof; hence, the musculature has to develop a force greater than the Stefan force. We return to this point in the next section where we derive a quantitative criterion for division of labor between the proboscis and pump.

### Importance of impulse of muscular force Π

The first integral of eqs (26) and (28) emphasizes the importance of the *impulse of muscular force*, ${\Pi }=-{\int }_{0}^{t}Fdt,$ which becomes the main characteristic of the musculature action:

30 $$\begin{array}{c}{Rectangular}\,{chamber}\\ \frac{{\Pi }{d}_{p}^{4}}{6\eta {L}^{4}}=-({{\rm{C}}}_{r}-\frac{W}{12L})(\frac{1}{{H}_{0}^{2}}-\frac{1}{{H}^{2}})+\frac{64}{3}\frac{{l}_{p}}{\pi {d}_{p}}{(\frac{W}{L})}^{2}(H-{H}_{0}){,}\end{array}$$

31 $$\begin{array}{c}Circular\,chamber\\ \frac{{\Pi }{d}_{p}^{4}}{6\eta {R}^{4}}=-({{\rm{C}}}_{{\rm{c}}}-\pi /8)(\frac{1}{{H}_{0}^{2}}-\frac{1}{{H}^{2}})+\frac{64}{3}\frac{\pi {l}_{p}}{{d}_{p}}(H-{H}_{0}),\end{array}$$

where *H*(*t*) = *h*(*t*)/*d*_*p*_, *H*_*0*_ = *H*(0).

These formulas explicitly show that the position of the pump roof *h*(*t*) at each time moment *t* depends only on the impulse of force (i.e., application of force over time), not the force itself. Only in a special case of a constant force, *F* = *const*, can impulse of force be factorized as ${\Pi }={\int }_{0}^{t}Fdt=F\cdot t$.

**Figure 7:**
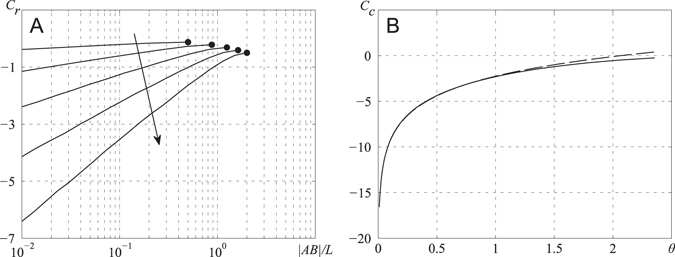
Dependence of constants *C*_*r*_ and *C*_*c*_ on size of pump openings |AB|/L and |AB|/2 R, respectively. (**A**) Rectangular chamber, dependence of *C*_*r*_ on |AB|/L for different W/L. The arrow indicates direction of increase of the W/L ratio, with uniform steps of 0.5, starting from 0.5 and ending at 2. Circles at the end of each curve correspond to limiting cases of pumps with W = AB. (**B**) Circular chamber, with dependence of *C*_*c*_ on *θ* = |AB|/(2 R); it tends to zero as θ → π. Dashed line is an empirical approximation of this dependence as *C*_*c*_ = 3.1 ln *θ* − 2.25.

In Fig. 7, the constants *C*_*r*_ and *C*_*c*_ entering eqs (26), (28), (30), and (31) have been calculated numerically to obtain their dependence on the size of the pump openings, i.e., |AB|/L for a rectangular and |AB|/(2 R) for a circular pump. Calculating eqs (27) and (29), the integrals over each triangle of the FEM mesh were calculated as the triangle area multiplied by pressure averaged over the three vertices of the given triangle, ${\iint }_{{\rm{\Delta }}}{\tilde{P}}_{c}dS\approx$ $\frac{1}{3}({p}_{1}+{p}_{2}+{p}_{3})S$, where *S* is triangle area, and *p*_*i*_, *i* = 1, 2, 3, are pressures numerically calculated at the triangle vertices by FEM in Matlab. For the rectangular pump, the *C*_r_ constant depends on two parameters, W/L and |AB|/L, whereas for the circular pump, the *C*_*c*_ constant depends only on a single parameter |AB|/(2 R). As the proboscis cross-section decreases, |AB|/L → 0 and |AB|/(2 R) → 0, these constants become larger in absolute value, reflecting significant hindrance of flow through thin apertures.

## Classification of energy dissipation mechanisms

The power provided by contractile muscular action of the insect to support fluid flow is partially. dissipated through viscous friction of the flowing fluid. This energy dissipation per second, $\dot{E}$, is calculated as $\dot{E}$ = *Fdh*/*dt*. From the proposed model, one can separate viscous losses caused by flow in the pump chamber, ${\dot{E}}_{c}$, and in the proboscis/esophagus, ${\dot{E}}_{p}$. Thus, total dissipation reads $\dot{E}=\,{\dot{E}}_{c}+{\dot{E}}_{p}$. Since the Pivnick & McNeil publication^57^, viscous losses in the proboscis have been considered the main source of energy dissipation. For a rectangular pump chamber, these losses are $\,{\dot{E}}_{p}={(dh/dt)}^{2}\frac{128\eta {l}_{p}}{\pi {d}_{p}^{4}}{(LW)}^{2}$ and for a circular chamber, these losses are ${\dot{E}}_{p}=$ ${(dh/dt)}^{2}\frac{128\eta \pi {l}_{p}}{{d}_{p}^{4}}{R}^{4}$. We, therefore, use this part, ${\dot{E}}_{p}$, as a reference and calculate total energy dissipation relative to this reference; i.e., we calculate the ratio $\dot{E}/{\dot{E}}_{p}\,$using eqs (26) and (28) as

32 $$\begin{array}{c}{Rectangular}\,{chamber}\,\quad \quad \quad \quad \quad \quad \quad \quad \quad {Circular}\,{chamber}\\ \frac{\dot{E}}{\,{\dot{E}}_{p}}=\,1+(\frac{3\pi {d}_{p}^{4}}{32{l}_{p}{h}^{3}})\,{(\frac{L}{W})}^{2}[\frac{W}{12L}-{C}_{r}],\quad \frac{\dot{E}}{\,{\dot{E}}_{p}}=\,1+(\frac{3{d}_{p}^{4}}{32\pi {l}_{p}{h}^{3}})\,[\frac{\pi }{8}-{C}_{c}].\end{array}$$

The constants *C*_*r*_ and *C*_*c*_ are always negative; therefore, the second term is always positive, adding to total energy dissipation. Thus, relative dissipation is always greater than one, $\dot{E}/{E}_{p} > 1$, and this statement holds for the uptake stroke as well as for the fluid-injection stroke when liquid is pushed through the esophagus.

Energy dissipation in the pump chamber is much less than that in the proboscis when the following inequalities hold true:

33 $$\begin{array}{c}{Rectangular}\,{chamber}\quad \quad \quad \quad \quad \quad \quad \quad Circular\,{chamber}\\ 1\gg (\frac{3\pi {d}_{p}^{4}}{32{l}_{p}{h}^{3}})\,{(\frac{L}{W})}^{2}[\frac{W}{12L}-{C}_{r}],\quad \quad 1\gg (\frac{3{d}_{p}^{4}}{32\pi {l}_{p}{h}^{3}})\,[\frac{\pi }{8}-{C}_{c}],\end{array}$$

These two sources of energy dissipation are equally important when the following conditions are satisfied

34 $$\begin{array}{c}{Rectangular}\,{chamber}\quad \quad \quad \quad \quad \quad \quad \quad Circular\,{chamber}\\ (\frac{3\pi {d}_{p}^{4}}{32{l}_{p}{h}^{3}})\,{(\frac{L}{W})}^{2}[\frac{W}{12L}-{C}_{r}]=1{,}\quad \quad \quad (\frac{3{d}_{p}^{4}}{32\pi {l}_{p}{h}^{3}})\,[\frac{\pi }{8}-{C}_{c}]=1\end{array}$$

Identifying the AB-opening with the diameter of the food canal of the proboscis, ${d}_{p},\,\,$and introducing new parameters

35 $$g={d}_{p}/L\,and\,\theta ={d}_{p}/(2R){,}$$

we can study eqs (34), rewriting them through the new functions ${f}_{r}(W/L,g)$ and ${f}_{c}(\theta )$ as:

36 $$\begin{array}{c}{Rectangular}\,{chamber}\quad \quad Circular\,{chamber}\\ {f}_{r}(\frac{W}{L}{,}g)=f{,}\quad \quad \quad \quad \quad \quad \quad {f}_{c}(\theta )=f{,}\end{array}$$

where

37 $$f=\frac{{l}_{p}{h}^{3}}{{d}_{p}^{4}},$$

38 $${f}_{r}(\frac{W}{L}{,}g)\equiv \frac{3\pi }{32}\,{(\frac{L}{W})}^{2}[\frac{W}{12L}-{C}_{r}(\frac{W}{L}{,}g)]\,{\rm{and}}\,{f}_{c}(\theta )\equiv \frac{3}{32\pi }[\frac{\pi }{8}-{C}_{c}(\theta )].$$

**Figure 8:**
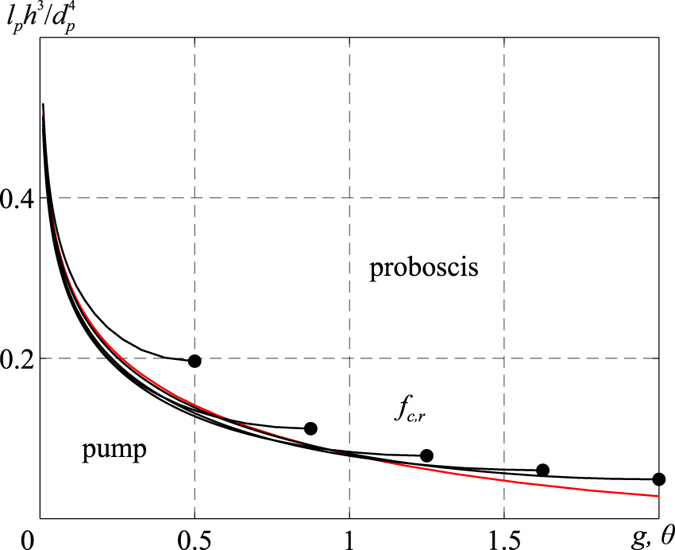
Classification of the pump–proboscis pair with respect to energy dissipation mechanisms. The sequence of curves is the same as that in Fig. 7. The red curve indicates a circular pump, and the black curves indicate rectangular pumps. All parameters used to calculate these black curves are identical to those in the caption of Fig. 7.

The right-hand sides of both equations (36) are the same; hence, the dissipation mechanism changes when physical parameters of the sucking pump and proboscis fall outside the boundaries given by the curves (36). These curves separate regimes of proboscis-limited energy dissipation from regimes of pump-limited energy dissipation. Figure 8 classifies these different regimes. For insects with physical parameters sitting above the *f*-curves, energy dissipation is associated with fluid flow through the proboscis. For insects with physical parameters sitting below the *f*-curves, energy dissipation is associated with fluid flow through the pump. As follows from this figure, when maximum displacement of the pump roof is about $h\sim {d}_{p}{(\frac{{d}_{p}}{{l}_{p}})}^{1/3}$, dissipation in the proboscis dominates dissipation in the pump chamber for any parameters *g* and *θ*.

To use this figure requires three steps: a) Calculate the geometrical parameters of the pump, such as W/L, *g*, or *θ*. b) Once these parameters are identified, draw the corresponding g or *θ* vertical line until it crosses the black or red boundaries. c) Calculate the ratio $\frac{{l}_{p}{h}^{3}}{{d}_{p}^{4}}$. If the calculated value is greater than the borderline in Fig. 8, then ${\dot{E}}_{p} > {\dot{E}}_{c}$; otherwise ${\dot{E}}_{p} < {\dot{E}}_{c}$. In Table 1, we list these parameters for our studied lepidopterans.

## Discussion

The intimate functional relationship of the proboscis and pump is illustrated by elongation of the proboscis over evolutionary time, which carries the need for increased pump musculature and thickened cuticular walls of the proboscis^38^. In fact, Bauder *et al*.^38^ were the first to emphasize that the suction pump must be considered in conjunction with the proboscis to understand the evolution of exceptionally long proboscises. Viewing the proboscis and pump as a functional complex permits inferences about pump size, extent of muscular development, proboscis length, and perhaps head size, and, consequently, feeding habits. Long-proboscis Lepidoptera typically have larger dilator muscles than do short-proboscis species^38^, as do fruit piercers compared with nectar feeders^58^. However, muscular development of the pump in the honey-feeding *A*. *atropos* is not markedly different from that of the nectar-feeding *M*. *sexta* ^41^, although our measurements indicate that the dimensions of their pumps differ.

Our results indicate that the majority of species with a long proboscis have to combat the frictional force associated with fluid flow through the proboscis. The data in Table 1 show that in all of our studied insects, except *Symmerista albifrons*, proboscis dissipation is the dominant dissipation mechanism. Figure 8 presents the categorization of insects for which proboscis dissipation ${\dot{E}}_{p}$ dominates pump dissipation ${\dot{E}}_{c}$. Although the sucking pump and musculature of these species are developed, these insects spend the majority of musculature energy to bring fluids to the pump by pushing it through the proboscis. For rectangular and circular pump chambers, proboscis dissipation is ${\dot{E}}_{p}={(dh/dt)}^{2}\frac{128\eta {l}_{p}}{\pi {d}_{p}^{4}}{A}^{2}=B{(dh/dt)}^{2}\eta$. Thus, for the insect to be efficient in fluid uptake, the proboscis and pump geometrical parameters defined by the constant $B=\frac{128\mu {l}_{p}}{\pi {d}_{p}^{4}}{A}^{2}$, as well as fluid viscosity, must be adjusted to the given muscular power. Taking the measured parameters from Table 1, we get $B=0.17\cdot {10}^{3}m$ and $765\cdot {10}^{3}m < B < 1070\cdot {10}^{3}m$ for *A*. *atropos* and *M*. *sexta*, respectively. The four orders of magnitude difference between the *B* factors explains why the head anatomy, including the musculature and pump, of *A*. *atropos* feeding on honey is not significantly different from that of *M*. *sexta* feeding on nectar. The muscular power of an insect should be proportional to ${\dot{E}}_{p}$ and, hence, should depend on the product *Bη*. Therefore, assuming that *A*. *atropos* has the same muscular power as that of *M*. *sexta* and the rate of pump chamber expansion *dh*/*dt* is the same for both insects, we can estimate the order of magnitude of honey viscosity *η*_*honey*_ that *A*. *atropos* is able to drink by equating the powers ${B}_{A.atropos}\cdot {\eta }_{honey}\approx {B}_{M.sexta}\cdot {\eta }_{nectar}\,$. This shows that ${\eta }_{nectar}/{\eta }_{honey}\sim {10}^{-4}$. Nectar is typically 10–100 times less viscous than honey^55, 56^. Therefore, *A*. *atropos* should be able to pull in liquids with viscosities about ten thousand times greater than those of nectar, without changing the anatomy of the pump and musculature.

Less attention has been given to lepidopteran proboscises too short to coil, perhaps because most known examples are of obscure nocturnal moths, particularly those of the families Notodontidae^59, 60^ and Geometridae^61^. Phylogenetically, the Notodontidae and Geometridae nest deep within the Lepidoptera with a coilable proboscis^62^, indicating selection for a secondarily reduced proboscis in certain taxa (e.g., *Phigalia strigataria* ^61^, *Clostera albosigma*, *Gluphisia septentrionis* ^59^, and *Symmerista albifrons*) while maintaining the longer, coilable option in related taxa (e.g., *Nadata gibosa*). Proboscis reduction is found in nearly all nine subfamilies of the Notodontidae^63^. We suggest that secondary reduction of the proboscis opened new niches for non-nectar feeding^59^, with new functionality embedded. Thus a short, noncoilable proboscis might have facilitated diversification of feeding habits within the Notodontidae in the same way as suggested for the lepidopteran proboscis generally^25, 64^. Members of family Notodontidae exhibit an impressive range of proboscis lengths and extraordinary diversification of proboscis surface sculpturing and sensilla morphology^63^. Not surprisingly, proboscis architecture has been a key source of characters in notodontid phylogenetic inference^63^. Notodontids, therefore, offer a rich opportunity for comparative investigations of feeding behavior, associated anatomy, and fluid mechanics of fluid-feeding insects. Feeding behavior among notodontid adults includes specialization on mud puddles^59^ and vertebrate wounds, perspiration, and lachrymal secretions^65^.

Differential proboscis and pump development among species suggests differential selection forces operating on feeding behavior. Selection pressures responsible for the short, dorsoventrally compressed proboscis of *S*. *albifrons* are enigmatic. Whether the adults actually feed or even take water is not known. Until 1982^59, 63^, notodontids with a short proboscis were not thought to feed as adults. Evidence suggesting that *S*. *albifrons* does not feed—short non-linked galeae ensconced within brushes of scales—is countered by strong circumstantial evidence in favor of fluid uptake: (1) intricate galeal surface sculpturing associated with capillarity^64, 66^, (2) a concave groove along the medial galeal margin, representing a food canal, (3) sensilla in this groove, (4) a well-developed sucking pump and complete set of associated muscles, (5) and occurrence of other species in the same family (Notodontidae) with short proboscises that are fully functional and permit uptake of large quantities of ion-enriched water^59^.

Data in Table 1 and Fig. 8 suggest that *S*. *albifrons* is able to switch the dissipation mechanism from pump to proboscis, provided it can bring the galeae together or move them apart on demand to control flow. For example, if the galeae are linked together forming a food canal of diameter 17.3 μm, the moth will have to spend most of its energy moving fluid through the proboscis; the *f*-factor is *f* = 2891. However, if the galeae are separated, the moth will not need to spend much energy on transporting fluid through the food canal; owing to capillarity, in a few milliseconds liquid will spontaneously fill the intergaleal gap up to the entrance of the cibarium^67^. High-speed X-ray imaging shows that a liquid meniscus, just after its formation at the edge of a capillary of 50 μm diameter, travels a distance of 250 μm in about 10 milliseconds^67^. Thus, capillary forces alone will ensure liquid delivery to the cibarial entrance, without involvement of the pump. Setting *l*_*p*_ = 0, we obtain an *f*-factor of zero, shifting the moth from the proboscis-dissipation category to the pump-dissipation category.

This interesting phenomenon of flipping the energy dissipation mechanism from the proboscis to the pump might have important physiological and behavioral implications. For example, some Notodontidae with short proboscises pass a significant amount of water by jetting it rapidly through the body^59, 60^. Over evolutionary time, this jetting mechanism might have been enabled by shortening the proboscis to significantly reduce energy dissipation of fluid transport through it. According to the dissipation criterion, a separated proboscis could functionally replace the cibarial valve. To accelerate flow, the moth must open the intergaleal gap; to decelerate flow, it must bring the galeae together. Adult Lepidoptera with a coilable proboscis routinely use behavioral strategies to ease physical and mechanical constraints on their feeding devices. For instance, by behaviorally separating the galeae apically, they can reduce frictional forces imposed on fluid uptake by a narrow food canal^28^ and they can manipulate the interlegular spacing by bending the proboscis to exert more control over fluid uptake and saliva delivery^25, 68^. Modifications of a noncoilable proboscis might have presented new opportunities for Lepidoptera to acquire trace nutrients from dilute fluids, such as mud puddles, some species even floating on water while drinking^59^. The ability to exploit dilute solutions in nature and extract minute quantities of nutrients and elements, such as sodium, is fundamental to reproductive success^60, 69^, perhaps even enabling multiple matings that otherwise would deplete the insect’s supply of nutrients.

## Conclusions

Coupling the sucking pump and proboscis into a united functional organ is important for understanding feeding habits of fluid-feeding insects in general and of Lepidoptera in particular. We hypothesized that in insects with a small ratio of chamber size to proboscis length, energy dissipation should be associated with the viscous drag of liquid moving through the proboscis (“proboscis dissipation”), whereas in insects with a large ratio of chamber size to proboscis length, energy dissipation should come from the viscous drag of liquid on the moving pump plunger (“pump dissipation”). This hypothesis is supported by the analysis of flow features inside the proboscis and pump.

We conducted a theoretical analysis of flow phenomena accompanying the suction stroke. A simple model of the pump chamber as a U-shaped rectangular or cylindrical dish with a movable plunger was used. The proboscis was modeled as a cylindrical tube. We classified insects with respect to the dominant dissipation mechanism (i.e., proboscis dissipation vs. pump dissipation) associated with viscous friction in the feeding organ. We showed that the description of dilator muscles originating on the head capsule and inserting on the flexible roof (plunger) of the sucking pump should be given in terms of impulse of force, *Π*, not force itself. Only in a special case when dilator muscles exert a constant force *F* on the plunger at each time moment, *t*, can the impulse of force be factorized as ${\Pi }={\int }_{0}^{t}Fdt=F\cdot t$. Impulse of force contains much more physiological information on functioning of the dilator muscles and, hence, requires more serious attention.

Two physical mechanisms of energy dissipation characterize the proboscis–pump complex. The first mechanism is the Stefan viscous adhesion of the plunger to the floor of the pump chamber. The second mechanism is related to viscous friction of liquid flowing through the proboscis. When coupled, these two mechanisms control division of labor between the proboscis and the sucking pump.

Movement of the plunger establishes a non-homogeneous pressure distribution in the pump: suction pressure closer to the esophagus is greater than that at the junction of the proboscis with the pump chamber. Therefore, dilator muscles nearest to the esophagus are under stronger tensile force, compared with those closer to the cibarium. The generated pressure gradient significantly depends on the rate of pull of the plunger. As a result, the pressure differential applied to the proboscis is not constant during the suction stroke. For long tubes, when energy dissipation in the pump is much smaller than that in the acquisition tube, the pressure differential mostly depends on the rate of the plunger pull, *p* ∝ *dh*/*dt*, and does not significantly depend on the current position of the plunger, *h*, at any given time moment *t*. However, at the first moment of the stroke, when distance between the plunger and chamber floor is small, h → 0, pressure differential is anomalously large, *p* ∝ h^−3^ *dh*/*dt* → ∞, implying that the force generated by the dilator muscles has to be extremely high, *F* ∝ h^−3^ *dh*/*dt*. However, the dilator muscles sense only the impulse of force, *Π*, which scales as *Π* ∝ *Ft*, and hence can be made finite at these small time intervals, *t*.

The second mechanism of energy dissipation is conveniently characterized by the power needed for insect musculature to move liquid through the proboscis: ${\dot{E}}_{p}=B{(dh/dt)}^{2}\eta$. The *B* factor depends on geometrical parameters of the proboscis and pump $B=\frac{128\eta {l}_{p}}{\pi {d}_{p}^{4}}{A}^{2}$. Therefore, fluid viscosity *Π* is not the sole determinant of the required muscular strength. To illustrate this statement, we compared the *B* parameters for *A*. *atropos* feeding on honey and *M*. *sexta* feeding on almost inviscid nectar. If the pump chamber of *A*. *atropos* is replaced with that of *M*. *sexta*, the insect would be able to pull in honey.

Further classification of insects with respect to the mechanism of energy dissipation is conveniently done by introducing two dimensionless parameters $f={l}_{p}{h}^{3}/{d}_{p}^{4}$ and *g* = *d*_*p*_/*L*. The demarcation in Fig. 8 specifies the groups of insects that dissipate their muscular energy mostly in transporting fluids through the sucking pump versus the proboscis. The derived diagram allows one to examine the fluid-mechanics constraints on evolutionary development of the feeding organ of insects. For insects with a large *f*-factor, musculature energy is mostly spent to combat viscous drag of fluids moving through the proboscis. For insects with a small *f*-factor, musculature energy is mostly spent to combat viscous drag of fluids moving through the pump. For example, in Lepidoptera, sucking-pump morphology and dilator musculature are similar across broad groups of insects that feed either on thin (water-like) or thick (honey-like) fluids. However, proboscis architecture, including its length to diameter ratio, varies significantly from species to species. Moreover, moths with unlinked galeae could use the action of alternately bringing the galeae together and separating them as a valve. This action will allow them to accelerate and decelerate fluid uptake on demand by flipping the dissipation mechanism from proboscis-limited to pump-limited. The hypothesis is testable through simple feeding studies to determine if the moths can alternately bring the galeae together and then separate them.

The anatomical features of the proboscis–pump complex in notodontids, representing coilable and noncoilable proboscises with well-developed sucking pumps, suggest a platform for interpreting evolution of adult lepidopteran feeding habits. The functional complex of proboscis and pump, together with variation in proboscis surface sculpturing and sensilla morphology^63^, requires integration with studies of actual feeding behavior to reach a more complete understanding of the evolution and diversification of lepidopteran feeding habits.

Fluid-feeding insects are characterized by a feeding organ that consists of a fluid-acquisition device and a sucking pump. The pump has similar architecture across many insects^70^, whereas the mouthparts vary significantly in shape and materials organization. We examined the simplest tube-like acquisition device, typified by the lepidopteran proboscis. Future studies are needed to explore the possible role that the sucking pump–proboscis pair has played as a possible key innovation fostering the enormous biodiversification of insects.
